# Mapping of Autogenous Saphenous Veins as an Imaging Adjunct to Peripheral MR Angiography in Patients with Peripheral Arterial Occlusive Disease and Peripheral Bypass Grafting: Prospective Comparison with Ultrasound and Intraoperative Findings

**DOI:** 10.1371/journal.pone.0112340

**Published:** 2014-11-18

**Authors:** Ann-Marie Bintu Munda Jah-Kabba, Guido Matthias Kukuk, Dariusch Reza Hadizadeh, Frank Träber, Arne Koscielny, Mustapha Sundifu Kabba, Frauke Verrel, Hans Heinz Schild, Winfried Albert Willinek

**Affiliations:** 1 University of Bonn, Dept. of Radiology, Germany; 2 University of Bonn, Dept. of Surgery, Germany; University of Washington, United States of America

## Abstract

**Background:**

Mapping of the great saphenous vein is very important for planning of peripheral and coronary bypass surgery. This study investigated mapping of the great saphenous vein as an adjunct to peripheral MR angiography using a blood pool contrast agent in patients who were referred for evaluation of peripheral arterial occlusive disease and bypass surgery.

**Methods:**

38 patients with peripheral arterial occlusive disease (21 men; mean age: 71 years, range, 44–88 years) underwent peripheral MR angiography using the blood pool contrast agent Gadofosveset trisodium. Apart from primary arterial assessment images were evaluated in order to determine great saphenous vein diameters at three levels: below the saphenofemoral junction, mid thigh and 10 cm above the knee joint (usability: diameter range: >3 and <10 mm at one level and >3.5 and <10 mm at a neighboring level). Duplex ultrasound was performed by an independent examiner providing diameter measurements at the same levels. Additionally, vessel usability was determined intraoperatively by the vascular surgeon during subsequent bypass surgery.

**Results:**

Mean venous diameters for MR angiography/duplex ultrasound were 5.4±2.6/5.5±2.8 mm (level 1), 4.7±2.7/4.6±2.9 mm (level 2) and 4.4±2.2/4.5±2.3 mm (level 3), respectively, without significant differences between the modalities (*P* = 0.207/0.806/0.518). Subsequent surgery was performed in 27/38 patients. A suitable saphenous vein was diagnosed in 25 and non-usability was diagnosed in 2 of the 27 patients based on MR angiography/duplex ultrasound, respectively. Usability was confirmed by intraoperative assessment in all of the 24 patients that received a venous bypass graft in subsequent bypass surgery. In 1 case, in which the great saphenous vein was assessed as useable by both MR angiography and duplex ultrasound, it was not used during subsequent bypass surgery due to the patients clinical condition and comorbidities.

**Conclusion:**

Simultaneous mapping of the great saphenous vein as an imaging adjunct to peripheral MR angiography with a blood pool contrast agent is an alternative to additive duplex ultrasound in patients undergoing subsequent peripheral bypass grafting.

## Introduction

The first bypass surgery with an autologous saphenous vein was carried out by Jean Kunlin in 1948 [1]. Nowadays bypass grafting has been fully established as a standard surgical treatment option in peripheral arterial occlusive disease (PAOD). An autologous saphenous vein is generally regarded as the material of choice for a femorodistal bypass [2]–[4], since it provides the best patency rates [4] and its anatomic position, its length and its wall strength make it suitable to be used as an arterial bypass graft [5]. This is particularly true if it is of good quality i.e. wide enough in diameter as well as free of varicosities, thrombotic segments and too many tributaries [6].

Since the 1970 s, the preoperative assessment of the saphenous vein regarding its anatomic configuration, venous abnormalities and pathologic changes has been proven successful in evaluating its suitability as a bypass graft in arterial reconstructions [7], [8]. In the 1980 s, the use of phlebography [9] was challenged by B-mode ultrasound [10], [11] being a procedure which is not invasive, prevents confusion concerning the discrimination of deep and superficial veins and last but not least renders more accurate information about the venous diameters [12], [13].

The advantages of the preoperative saphenous vein mapping, i.e. reduction in morbidity, operation time and hospital stay [13], have been evaluated in several studies on coronary artery grafts [14], [15] as well as peripheral bypass surgery [16], [17].

The major quantitative parameter which has to be determined during venous mapping and has been identified as the predominant technical factor in predicting primary patency, primary assisted patency and secondary patency is the internal venous diameter [4]. In this context the superiority of veins having a larger diameter (>3.5 mm) over narrow ones (<3 mm) is well established [4], [18]. However, Wengerter et al. demonstrated that grafts having a diameter of 3.5 mm as well as grafts which have a diameter of 3.0 mm and are at the same time not longer than 45 mm, present similar patency rates compared to grafts which have a diameter of at least 4.0 mm [19]. This highlights the point that the decisive factor for the short- and long-term success of the bypass surgery is the suitability of the venous conduit, which may be even more important than the quality of the arteries.

B-mode ultrasound has been established as the gold standard in the preoperative mapping of the great saphenous vein (GSV) [20]. However, this noninvasive procedure is highly operator dependent, very time consuming, especially in patients who have already undergone venous surgery, and has technical limitations in patients with edema, obesity and ulcerations.

Contrast-enhanced magnetic resonance angiography (CE-MRA) is considered an alternative to digital subtraction angiography (DSA) in the diagnosis and assessment of patients with peripheral arterial occlusive disease [21], [22] and increasingly replaces DSA in the imaging of the arterial vascular system [23]. However, spatial resolution is limited with standard extracellular Gadolinium chelates during “first pass” imaging. Recently, a contrast agent, with a reversible albumin binding and an extended intravascular retention and contrast enhancement [24], a “blood pool contrast agent (BPCA)”, was approved by the U.S. Food and Drug Administration (FDA). During “first pass” it offers an image quality which, depending on the dose, is at least as good as that of standard extracellular contrast agents [25]–[27]. In addition to first pass MR angiography, blood pool contrast agents allow contrast enhancement during an “equilibrium phase” (i.e. steady state) due to the lack of a relevant extravasation of the contrast agent into the interstitial space. Providing an imaging window of up to 60 minutes [28], the equilibrium phase facilitates the acquisition of much higher spatial resolution images without a significant loss of vessel-to-background contrast [28]–[30]. Steady state MRA with the blood pool contrast agent Gadofosveset trisodium in patients with peripheral arterial occlusive disease was found to render better results than first pass imaging alone [31] and leads – as an add-on – to the simultaneous visualization of the venous system [30]. The additional visualization of veins to that of arteries is inherent to steady state MRA and requires neither a second contrast administration nor any other change of examination parameters. As early as in 1998, Grist et al. indicated the benefits which might be drawn from the simultaneous visualization of arteries and veins in the steady state imaging with Gadofosveset [29]. Today, preliminary data supports the hypothesis that this approach allows for MR-venography as an add-on to the MRA of the arterial system [32]–[34]. Gadofosveset-enhanced MR-imaging has already been proven successful in the detection of thromboembolic processes. Deep venous thrombosis [35] as well as collateral pathways in patients suffering from a massive thromboembolic occlusion of the central veins were reliably detected; the latter could even be achieved in a way better than conventional X-ray-based phlebography [30].

The basis of this work was the hypothesis that peripheral MRA in the steady state using a blood pool contrast agent to image the size and quality of great saphenous veins would be a useful adjunct to arterial phase MRA to improve the performance of peripheral bypass surgery in patients with PAOD. Thus, the purpose of this study was to prospectively determine the accuracy of high-spatial-resolution steady-state MR angiography using the blood pool contrast agent Gadofosveset trisodium in comparison to color-coded duplex ultrasound (DUS) as the standard of reference in order to assess the usability of autologous saphenous veins for subsequent peripheral bypass surgery. Results were correlated with intraoperative findings.

## Methods

### Ethics Statement

The study protocol was approved by the institutional ethic committee of the University Hospital Bonn, Germany (approval number: 132/07). All patients provided written informed consent.

### Patients

**Figure 1:**
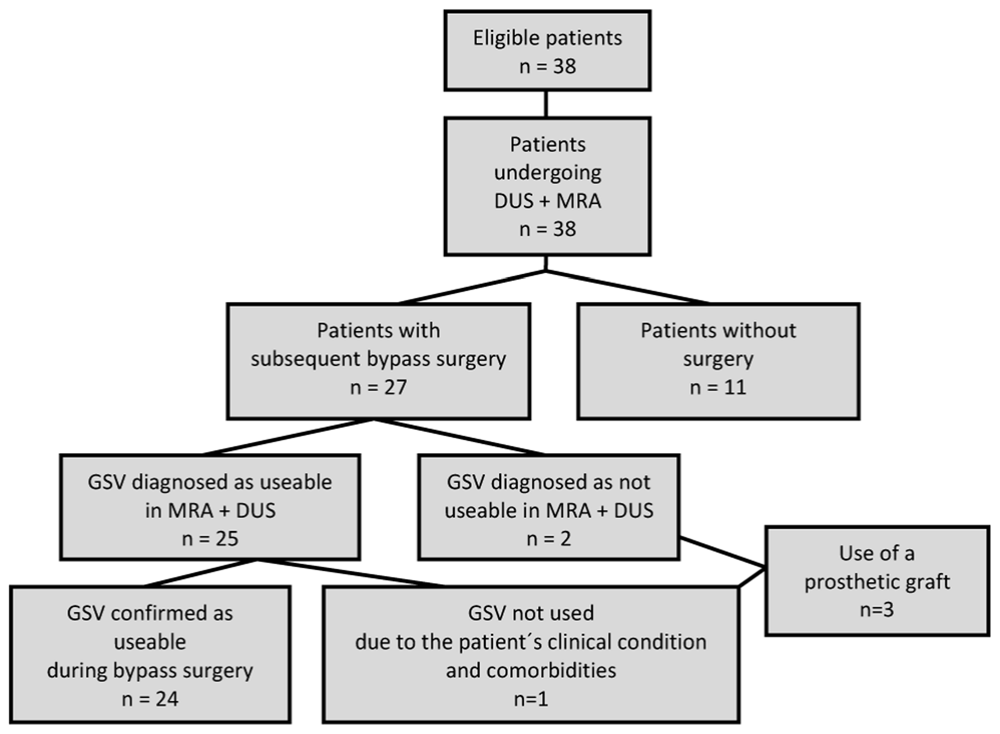
Flowchart portraying the study design.

This prospective, intraindividual comparative study included 38 consecutive patients (mean age, 71 years; range, 44–88 years) (21 men, 17 women) (table 1) with peripheral arterial occlusive disease who underwent 3D-enhanced MR angiography in preparation for or evaluation of a peripheral (femoropopliteal, popliteocrural or femorocrural) bypass surgery (figure 1).

**Table 1:** Clinical and demographic characteristics of the study population.

	PAOD stage II b	PAOD stage III	PAOD stage IV
number of patients	13	9	16
age range - years	57–78	45–88	56–85
male sex - no.	7	3	11

All patients were of white ethnicity.

Clinical inclusion criteria were PAOD stage IIb-IV (Fontaine classification) and indication for MRA. Exclusion criteria were contraindications for 3D contrast-enhanced MR angiography (e.g. allergy, metallic implants that are incompatible with MR imaging including pacemakers). The indication for MRA was defined clinically by the vascular surgeons. Every patient who was enrolled into the study also underwent DUS. 3D contrast-enhanced MR angiographic and ultrasound examinations were carried out in random order. MR-angiographic venous mapping was performed as an adjunct to the BPCA-MRA which was done in order to assess arterial occlusive disease. A possible surgical intervention was not delayed for any patient because of his or her participation in this study.

### MR Angiography

**Figure 2:**
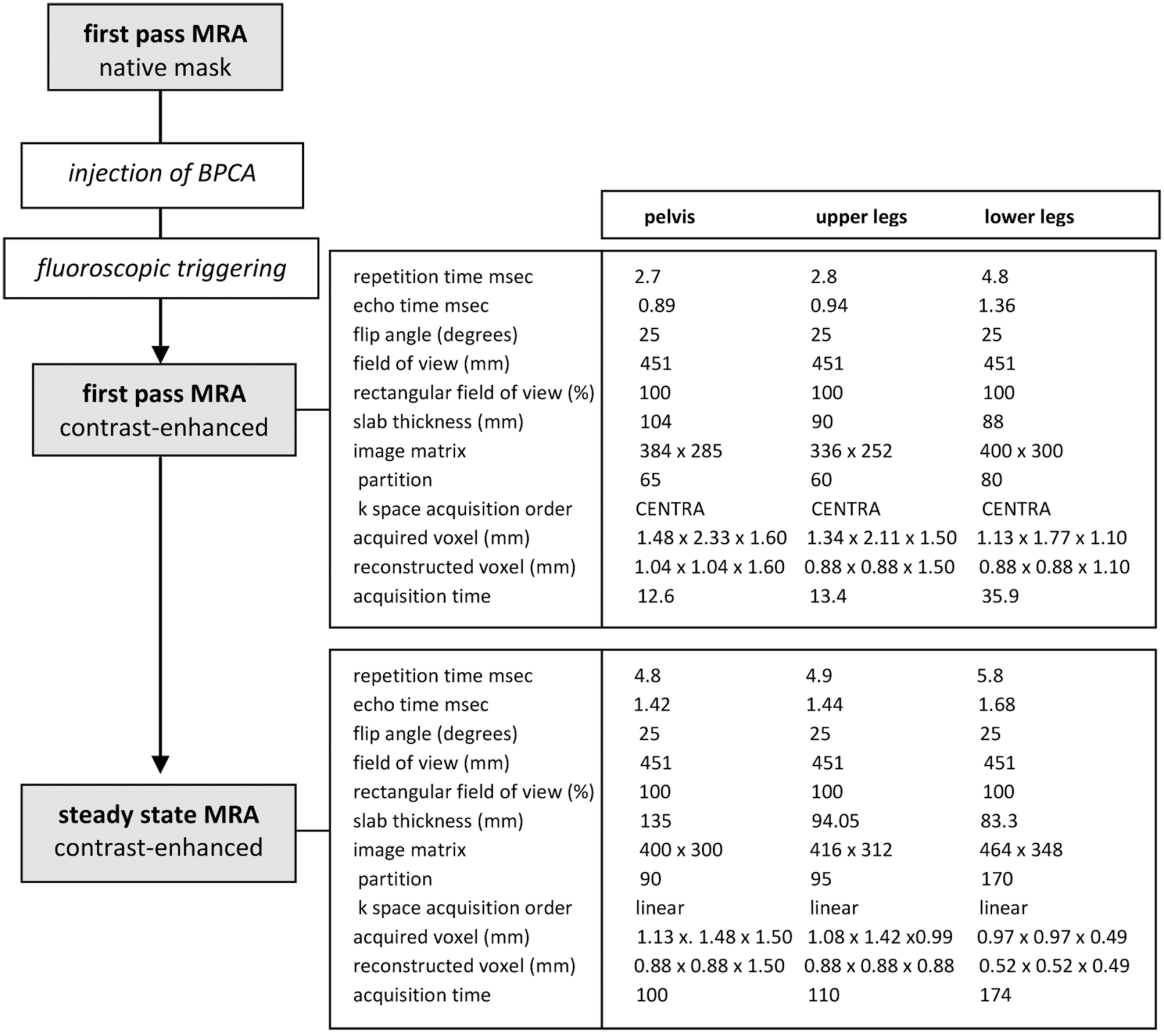
MR-angiographic protocol. Sequence flow of the combined first-pass and steady-state MR angiographic protocol and technical parameters of T1-weighted gradient-echo sequences for first-pass and steady-state MR angiography.

MR imaging was performed as previously described [31]: A 1.5-T whole–body imager (Achieva; Philips Healthcare) (maximum gradient amplitude, 33 mT/m; slew rate, 200 T/m/sec) was used to acquire three-dimensional contrast-enhanced MR angiographic sequences of the vasculature. While images of the lower legs were obtained using a commercially available flexible four-channel phased-array coil (Philips Healthcare, Best, the Netherlands), an integrated body coil served for image acquisition of the upper legs and pelvic region. Using a biphasic injection protocol, Gadofosveset trisodium was administered with an automatic power injector (Spectris; Medrad Europe, Beek, the Netherlands) at a flow rate of 1.2 ml/sec followed by a 25 ml saline flush at a flow rate of 0.6 ml/sec. As soon as the contrast medium was detected at the level of the common iliac arteries by means of fluoroscopic triggering, the acquisition of first pass images was started. The imaging protocol as well as technical parameters of the T1-weighted gradient-echo sequences that were used for first-pass and steady-state MR angiography are shown in figure 2 [31], [35]. Steady-state imaging followed first-pass imaging 4 minutes after contrast injection [36].

### Contrast Agent

Gadofosveset trisodium (Vasovist, Bayer Healthcare, Leverkusen, Germany; discontinued, now available in the US and Canada as Ablavar, Lantheus Medical Imaging, N. Billerica, MA) was the first intravascular contrast agent approved for use with MRA in the European Union, Switzerland, Turkey, Canada and Australia [37]. In 2008 it became the first contrast agent specifically approved for MR angiography in the US and Canada by the FDA in order to investigate aortoiliac occlusive disease. Due to an additional diphenylcyclohexyl group, this gadolinium-based contrast agent strongly but reversibly binds to human serum albumin resulting in a higher relaxivity (r1 = 19 L *mmol^−1 ^sec^−1^ at 1.5 T and 37°C in plasma) and an extended plasma half-life as compared to standard Gadolinium-containing contrast agents. A dose of 0.03 mmol/kg has been proven safe and effective for imaging of peripheral vascular disease [38] and was applied in all patients in our study.

### Image analysis of MR Angiography

**Figure 3:**
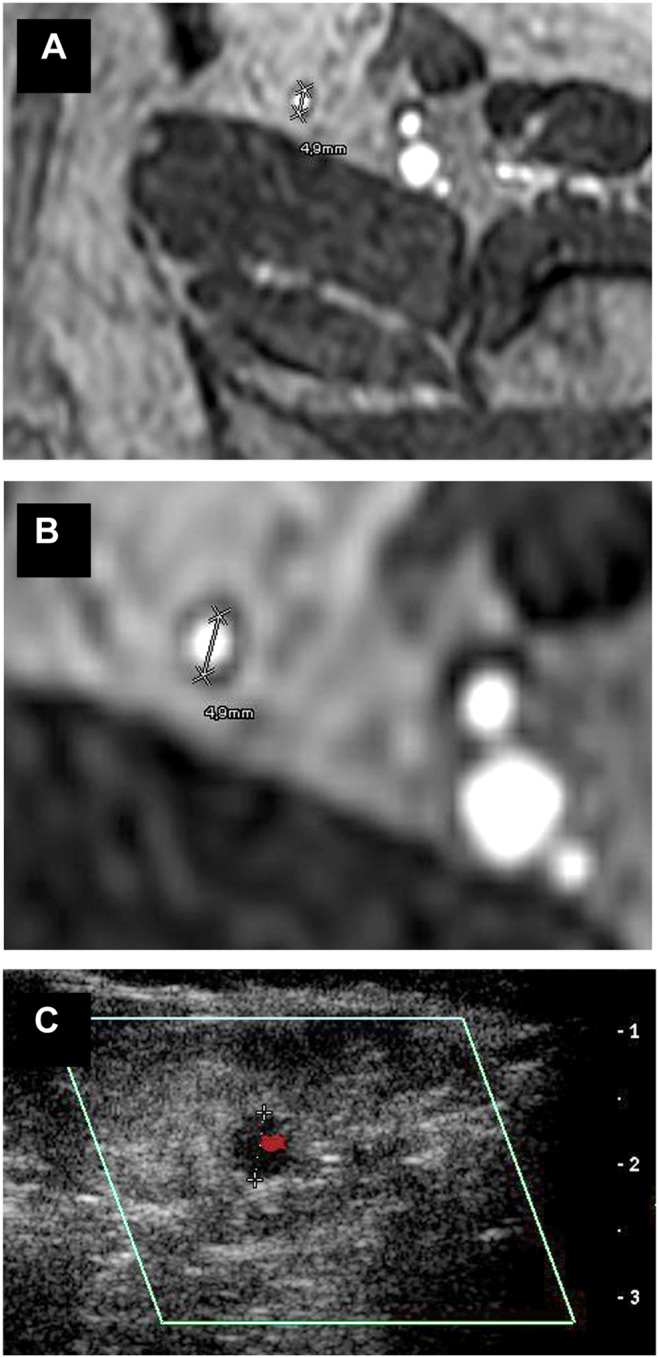
MR-angiographic and duplex sonographic images of the great saphenous vein. Magnetic resonance imaging (BPCA-MRA) and color-coded duplex sonography in the proximal level of the left GSV of a 63 year old female patient who suffered from PAOD stage III and was referred to the radiological department for assessment of the arterial status prior to a proposed bypass surgery. (a, b) Axial multiplanar reformat of contrast-enhanced T1-weighted gradient-echo images during the steady-state. (c) Axial color-coded duplex sonography.

**Figure 4:**
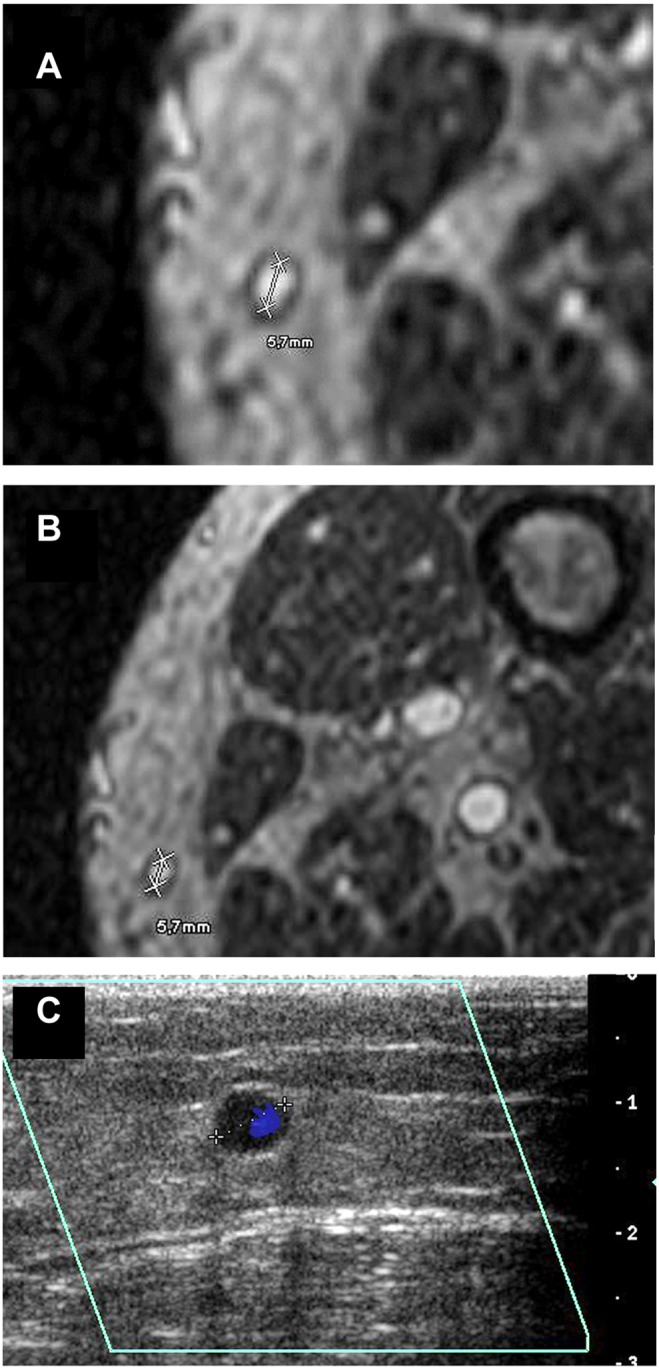
MR-angiographic and duplex sonographic images of the great saphenous vein. Magnetic resonance imaging (BPCA-MRA) and color-coded duplex sonography in the distal level of the left GSV in a 69 year old male patient with PAOD stage IV. (a, b) Axial multiplanar reformat of contrast-enhanced T1-weighted gradient-echo images during the steady-state. (c) Axial color-coded duplex sonography.

**Figure 5:**
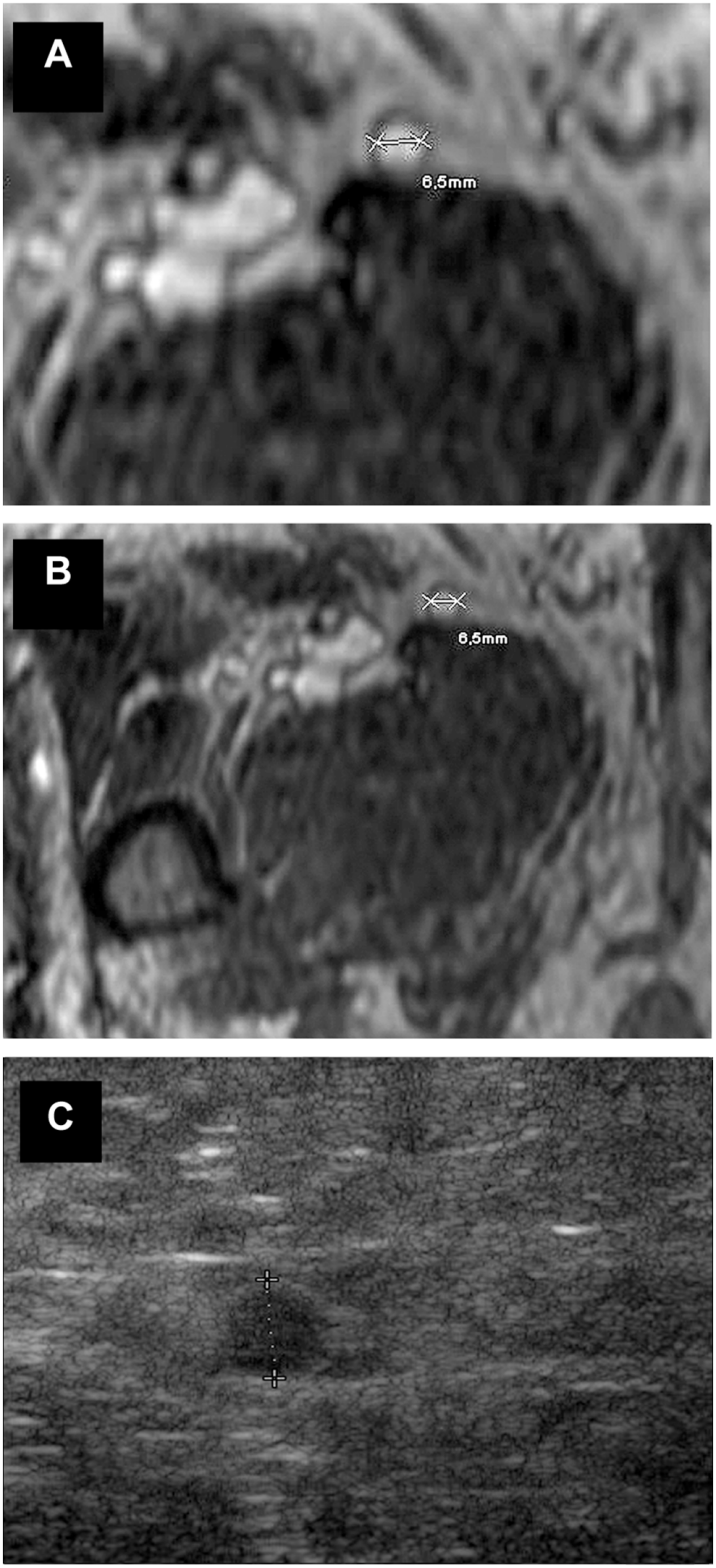
MR-angiographic and duplex sonographic images of the great saphenous vein. Magnetic resonance imaging (BPCA-MRA) and color-coded duplex sonography in the proximal level of the left GSV of a 69 year old male patient who suffered from ulcerations of the lower leg. (a, b) Axial multiplanar reformat of contrast-enhanced T1-weighted gradient-echo images during the steady-state. (c) Axial color-coded duplex sonography.

The maximum venous vessel diameter of the GSV (area of enhancing vessel lumen) was measured on cross-sectional images perpendicular to the flow axis by one radiologist (10 years of experience in vascular radiology) on multiplanar reformats of steady-state MR angiograms on a post-processing workstation (Viewforum; Philips Healthcare, Best, Netherlands) (figure 3–5). Each vein was measured at three levels: at the level just below the groin, at the level of the mid-thigh and 10 cm above the knee joint. To account for both the influence of the graft diameter on the patency rate and the influence of the graft length on the minimal diameter regarded as useable, [4] we decided to rate veins suitable which offered a diameter of at least 3.5 mm at one level and simultaneously a diameter of at least 3.0 mm at a neighboring level. In accordance to clinical practice we defined an upper limit of 10 mm. Nodular varicosities as well as other venous pathologies like thrombosis were exclusion criteria for use in bypass surgery. In order to minimize intraindividual measuring variability, three independent measurements were noted in each of the defined points and the mean was taken for comparison. Moreover a fixed magnification factor of 4 was applied. The reader was blinded regarding the patientś names, clinical histories and the results of other diagnostic procedures, including color-coded duplex sonography.

### Color-coded duplex sonography

Examinations were performed with the patient in supine position. A HD 11 ultrasound system (Philips Healthcare, Best, the Netherlands) with a 5–10 MHz ultrasonographic transducer was used to visualize the saphenous vein in transverse scans. Starting from the groin, the main trunk was tracked distally to the knee joint. Analogously to the MR angiographic image analysis, three independent measurements were taken from the cross-sectional scan at each level (figure 3–5) and the mean value was calculated. The reader of the DUS was blinded with regard to the results of the MRA.

### Intraoperative Evaluation

**Figure 6:**
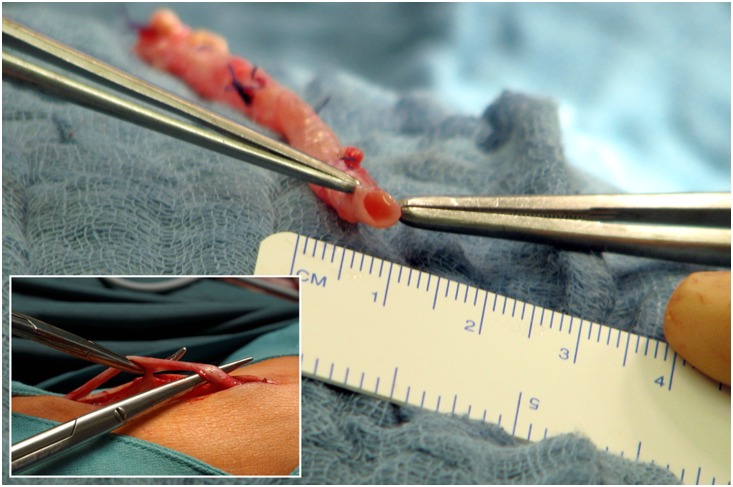
Intraoperative measurement of the dissected great saphenous vein.

The preparation of the GSV was carried out through an inguinal incision and a few auxiliary incisions along the saphenous vein. The vein was dissected and measured with a ruler as seen in figure 6. Branches were ligated, the vein was cut to size if necessary and anastomosed at its proximal and distal ends. Absolute values were not compared statistically because measurements were performed on dissected veins that are not in the same physiological condition as in vivo.

### Statistical analysis

Students t-test for paired samples was used to test the differences between the diameters measured by DUS and MR angiography based on a venous segment level. A *P* value of less than 0.050 was considered to indicate statistical significance. Correlation between the MR-tomographic and sonographic data was analyzed using Pearsońs correlation coefficient. All statistical analyses were performed with the Statistical package for Social Sciences (SPSS) version 17.0 (SPSS, Chicago, Il, USA).

## Results

3D steady-state MR angiography was successfully completed in 38 patients (76 assessable legs). Legs, which offered no measureable saphenous vein due to stripping of the saphenous vein (14/76), amputation at the level of mid-thigh (1/76) and absent definability in MRA as well as in sonography (no available level for comparison) (3/76) were excluded. Finally a total of 58 veins were available for intraindividual comparison at level 1, 54 veins for comparison at level 2, and 49 veins for comparison at level 3. The difference between the number of measurements available at the different levels is due to the fact that in some cases a segment (i.a. a level) of the vein was not included in the scanned volume (5/13) or certain levels were not clearly definable in MRA and/or DUS (8/13). All segments containing the measuring points were satisfactorily displayed on steady-state MR angiography as well as with DUS. Altogether, 161 comparative measurements were performed (Table S1).

**Figure 7:**
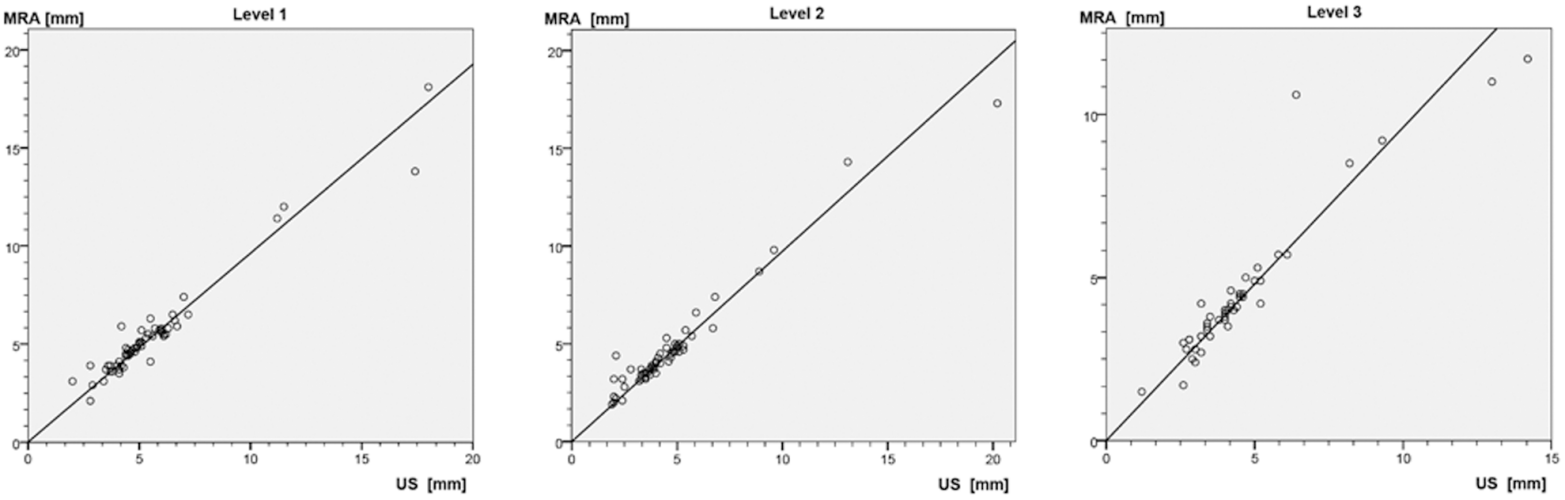
Correlation between MR-angiographic (MRA) and duplex sonographic (DUS) values in the three levels. Pearson correlation coefficients R^2^: level 1: 0.989; level 2: 0.986, level 3: 0.973.

Mean venous diameters in MRA/DUS for level 1, 2 and 3 were 5.4±2.6 mm/5.5±2.8 mm, 4.7±2.7 mm/4.6±2.9 mm and 4.4±2.2 mm/4.5±2.3 mm respectively, without significant differences between the two modalities (*P* = 0.207/0.806/0.518, df = 57/53/48) (figure 7). The venous diameters measured were higher on steady-state MR angiography than on color-coded duplex sonography in 54 out of 161 measurements (33.54%) (overestimation) and lower on 82 out of 161 measurement (50.93%) (underestimation). In 25 out of 161 measurements (15.53%) the two methods yielded identical values. The mean values of the positive and negative differences (MRA-DUS) are 0,52±0,67 mm and −0,45±0,59 mm, respectively, with an overall mean of differences (MRA-DUS) of −0,06±0,72 mm.

27 of the 38 patients underwent subsequent bypass surgery. In all of these 27 patients, the MR-angiographic assessment and the independent sonographic evaluation showed the same results regarding the suitability of the vein which was considered as bypass conduit. A suitable saphenous vein was diagnosed in all of the 24/27 patients that received a venous bypass conduit. Intraoperative findings confirmed usability in these 24 patients (13 femoropopliteal, 6 popliteocrural and 5 femorocrural bypasses). Non-usability was found in 2/27 patients based on MRA/DUS: In one of these patients the diameter of the respective saphenous vein was too small, the second patient had nodular varicosities. A prosthetic graft was used for reconstruction in both patients. In 1/27 patients a prosthetic graft was utilized despite the usability of the great sapehnous vein based on MRA and DUS findings. This decision was taken by the surgeons due to the patients clinical condition and comorbidities (including coronary heart disease) in order to minimize the duration of the operation as well as to save the saphenous vein for a potential coronary bypass surgery. In 11 patients no bypass surgery was performed.

## Discussion

Patients suffering from PAOD often present with concomitant venous disease [39].

In some patients that are evaluated for bypass surgery, veins or parts of them have already been removed due to varicosities or previous arterial surgery. Furthermore, the correct identification of a suitable vein helps to prevent unnecessary large dissections in already ischemic limbs [5], [40] and thereby reduces postoperative leg morbidity [13]. As such, the assessment of the usability of veins as a bypass graft is an indispensable part of the preoperative workup [41].

Our study demonstrates the diagnostic accuracy of MRA with a BPCA in comparison to DUS in the assessment of the luminal diameter of the GSV. Venous mapping was included in the MR-angiographic examination for arterial stenosis grading without the need of a change of examination parameters or any additional diagnostic step in the patient management. We compared sonographic and MR-angiographic measurements and tested the hypothesis that both modalities provide the same results concerning the usability of the GSV as a bypass conduit.

The visualization of the veins in the equilibrium phase of the MRA with Gadofosveset trisodium offered reliable results with regard to the luminal diameter of the GSV in our study population. Statistical analysis showed no significant differences between sonographic and MR-angiographic measurements. Usability (25/27) and non-usability (2/27) were diagnosed consistently by DUS as the standard of reference and MRA. Additionally, usability was verified by the intraoperative assessment of the vein in all patients that received a venous bypass conduit in subsequent bypass surgery.

MRA with the BPCA Gadofosveset trisodium has been proven to be safe, well-tolerated and highly efficient in patients with vascular disease [36], [42]. In 2000, a new disease which is now called nephrogenic systemic fibrosis (NSF) was first described [43]. In 2006, a link between NSF and the exposure to gadolinium-based contrast agents was observed [44], [45]. NSF is very rare and limited to patients with renal impairment [46], [47]. Following studies indicated that NSF almost exclusively occurs after administration of linear, non-ionic gadolinium-based contrast agents [44], [45]. Moreover a prolonged exposure to the contrast agent due to renal insufficiency is suggested as a factor in the pathogenesis of NSF [44], [45]. Although Gadofosveset trisodium is a gadolinium-based contrast agent with a linear structure and an extended plasma half life, so far no unconfounded cases of NSF after administration of a BPCA have been reported [44]. One reason for this might be the high efficiency of Gadofosveset trisodium which results in the use of a much lower Gadolinium concentration as compared to extracellular contrast agents [44]. Nevertheless, a responsible use of gadolinium-based contrast agents in patients with renal impairment is still obligatory.

The sequences needed for first pass and steady state MRA with a BPCA can be easily applied at any MR imaging system and allow for both arterial stenosis grading and the evaluation of venous diameters within the same scan with just a single-dose of contrast agent [35]. Traditionally, venous enhancement was considered a relative drawback of first-pass as well as of steady-state imaging because of impairment of arterial delineation [29], [30]. However, modern steady-state imaging with its increase in spatial resolution and the possibility to acquire isotropic voxels has proved to allow for a distinct analysis of arteries and veins without overlay [30], [31]. Our study lines with previous results that prove the potential of simultaneous visualization of arteries and veins, which was initially postulated in 1998 in view of examining the entire vasculature of the lower extremity in a single imaging procedure [29].

The accuracy of CE-MRA with a BPCA in arterial stenosis grading as compared with digital subtraction angiography (DSA) has already been demonstrated [31], [48]–[50]. Yet, few studies have investigated the inherent potential of the simultaneous visualization of arterial and venous structures in steady state imaging [33]–[35]. A recent study by Hadizadeh et al. showed that BPCA-MRA can reliably detect incidental venous thrombosis in patients undergoing peripheral MRA because of suspected PAOD [35]. Having demonstrated the reliability of BPCA-MRA in the venous diameter measurement and thus the assessment of the GSV as a bypass conduit, our findings support the usability of BPCA-MRA for simultaneously grading arterial stenosis and visualizing the peripheral veins of the lower extremities in a diagnostically conclusive way.

**Figure 8:**
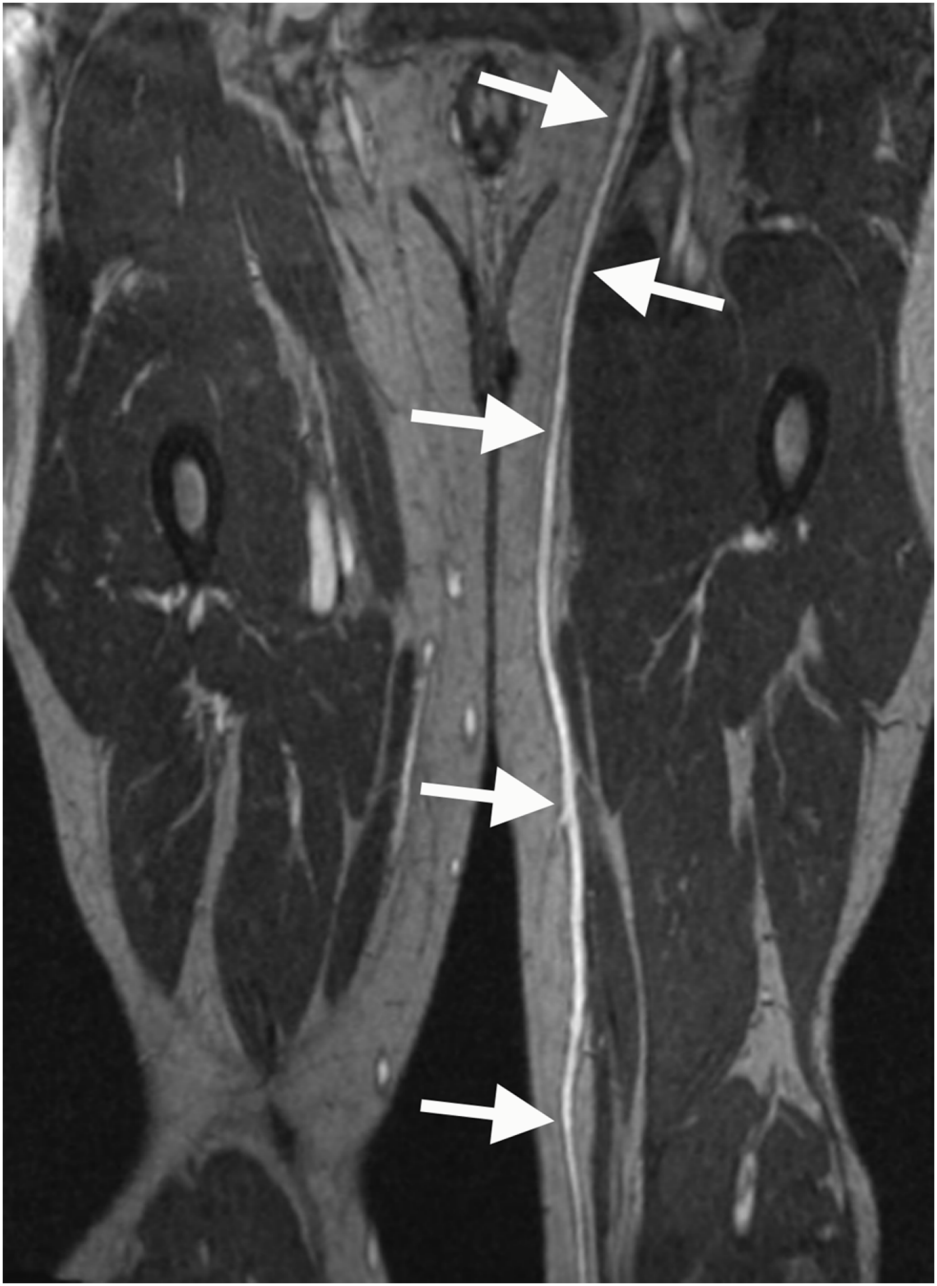
Multiplanar reformat of an MR-angiographic image of the great saphenous vein. The GSV displayed as a curved multiplanar reformat of high-spatial-resolution contrast-enhanced T1-weighted gradient-echo images during the steady-state of a 57 year old male patient suffering from PAOD stage III and thus being evaluated for bypass surgery.

Our study has several limitations. In the first place, the comparison of the mean venous diameters for the three levels as well as the comparison of the single measurements point out, that MRA in comparison to DUS tends to somewhat over- or underestimate the vessel diameter. However, the fact that the diagnosis of usability versus non-usability by MRA and DUS was consistent in all cases indicates that these slight differences of BPCA-MRA and DUS can practically be neglected. Still, it has to be taken into consideration that a larger study population will increase statistical power. Secondly, the fact that the MRA was carried out as a routine examination for the evaluation of the arterial system, in some cases resulted in the MR technologist accidently excluding the saphenous vein from the scan volume, leading to a reduced number of available segments for comparisons of level 2 (54/76) and 3 (49/76) diameters in comparison to level 1 (58/76). In relation to DUS [19], an inherent limitation of the MR-angiographic examination is the inability to preoperatively mark the course of the veins on the skin as a guide for bypass surgery [10]. However, similar to conventional venography (phlebography), MRA offers a ’roadmap’ of the entire vascular system of the lower extremity for treatment planning [21], especially if curved multiplanar reformats are reconstructed (figure 8). In combination with the option to examine the vascular system in different planes, diagnostic analysis and treatment planning are facilitated [21] even in cases of difficult sonographic evaluation due to obesity, previous surgical interventions, multiple collaterals or varicosities. Moreover, in contrast to phlebography, the depth of the saphenous vein as well as the distinction between deep and superficial veins is clearly evident. Yet, the currently limited access to post-processing workstations might hamper the surgeons first-hand preoperative look at the saphenous vein.

In this context, the benefits of DUS are well known: it is a noninvasive, relatively cheap, mobile and adjustable procedure free of ionizing radiation and contraindications and risks related to strong magnetic fields [21]. Keeping this in mind, BPCA-enhanced MRA for assessment of the GSV may appear a rather expensive and inconvenient approach in relation to DUS. However, the MR-angiographic assessment of the GSV vein is not proposed as a separate examination but as an adjunct to MRA which is anyway implemented for evaluation of the run-off arteries. Since the ’add-on’ only relates to image analysis and not to the MRA procedure itself, most of the advantages of DUS mentioned above are outweighed. The duration of performing the bilateral image analysis of the GSV by the radiologist, which in our cohort was between 3 to 8 minutes, is actually the only additional time and cost factor caused by the MR-angiographic evaluation of the GSV. The bilateral sonographic examination took between 5 to 15 minutes without calculating the patient or equipment transport required. Moreover, the MRA technique is less examiner-dependent than DUS, can be documented in a standardized way and can be reanalyzed at any time. Furthermore, the ability to assess arteries and veins at the same time within one single diagnostic test, reduces the number of examinations, simplifies preoperative diagnostics as well as patient management and may thus shorten the length of the hospital stay [35].

Another limitation of this study was due to the surgical practice that a vein identified as unusable by DUS did not undergo an additional intraoperative evaluation. An intraoperative evaluation of such veins would generally not yield an assessment differing from the ultrasonographic evaluation, which renders them unsuitable for bypass surgery. Its intraoperative exploration would therefore cause unnecessary trauma to the patient.

A final limitation of our study is the fact that in the bypass surgeries performed, the use of the GSV was often not limited to its supragenual segment. In several cases the length of the bypass required resulted in the additional intraoperative dissection and use of the infragenual segment of the GSV. Intraoperatively, in three patients the diameter of the infragenual segment of the vein turned out to be too small to be used as a bypass conduit. In these cases the GSV of the contralateral lower leg, the small saphenous vein of the contralateral lower leg and a prosthetic graft were used respectively to complement the supragenual GSV in order to acquire a bypass conduit which is long enough. This observation does not hamper the validity or significance of our results but retrospectively highlights that an extension of our preoperative mapping to the infragenual GSV could have presented useful additional information for the surgeons.

In conclusion, the MR-angiographic assessment of the suitability of the saphenous vein as a bypass conduit corresponded with the sonographic evaluation in all of the patients who underwent peripheral MRA and subsequent bypass surgery. With BPCA-MRA, a reliable assessment of the venous diameter of the GSV is possible and shows no significant differences in comparison to DUS as the standard of reference. Consequently, in patients with PAOD who in the preoperative workup undergo peripheral MRA to evaluate the arterial status, the inherent MR-angiographic imaging of autogenous saphenous veins can offer additional information regarding the suitability of the veins as a bypass conduit without the need of an additive ultrasound examination.

## Supporting Information

Table S1 **Diameters of the great saphenous vein.**
(DOC)
