# Supplementary material for: Mapping of Autogenous Saphenous Veins as an Imaging Adjunct to Peripheral MR Angiography in Patients with Peripheral Arterial Occlusive Disease and Peripheral Bypass Grafting: Prospective Comparison with Ultrasound and Intraoperative Findings
Source: PLoS One. 2014 Nov 18;9(11):e112340. doi: 10.1371/journal.pone.0112340 (PMC4236072; doi:10.1371/journal.pone.0112340)
Supplement: Table S1 — Diameters of the great saphenous vein. (DOC) [file pone.0112340.s001.doc]

**Table S1.** **Diameters of the great saphenous vein.**

|  |  | right GSV (in mm) | | | | | | left GSV (in mm) | | | | | |
| --- | --- | --- | --- | --- | --- | --- | --- | --- | --- | --- | --- | --- | --- |
|  | graft | MRA | | | DUS | | | MRA | | | DUS | | |
| 1 | v | 3.6 | 3.8 | 3.7 | 3.8 | 3.9 | 3.8 | stripping | | | | | |
| 2 | v | 4.7 | 4.5 | --- | 4.7 | 4.2 | 4.4 | 5.4 | 5.0 | 4.5 | 5.6 | 4.9 | 4.5 |
| 3 | v | 4.8 | 3.5 | 3.2 | 4.9 | 3.5 | 3.5 | 4.9 | 3.8 | ni | 5.1 | 3.8 | 3.0 |
| 4 | v | 5.7 | 4.8 | 3.1 | 5.9 | 5.0 | 2.8 | 5.6 | 4.9 | 4.0 | 6.0 | 5.0 | 4.0 |
| 5 | v | stripping | | | | | | 13.8 | 8.7 | 8.5 | 17.4 | 8.9 | 8.2 |
| 6 | v | 6.5 | 5.3 | 4.1 | 7.2 | 4.5 | 4.4 | 6.2 | 5.4 | 4.5 | 6.6 | 5.7 | 4.6 |
| 7 | v | stripping | | | | | | 4.5 | 3.9 | 4.2 | 4.4 | 3.8 | 4.2 |
| 8 | v | 5.5 | 4.6 | 4.9 | 5.4 | 4.9 | 5.2 | 5.3 | 4.5 | 4.9 | 5.3 | 4.7 | 5.0 |
| 9 | v | 3.7 | 3.5 | 4.0 | 4.1 | 4.0 | 4.0 | 4.1 | 4.0 | 3.5 | 4.1 | 4.0 | 3.4 |
| 10 | v | 5.9 | 4.9 | 4.2 | 6.7 | 5.3 | 5.2 | 5.4 | 4.3 | 4.2 | 6.1 | 4.1 | 3.2 |
| 11 | v | 5.0 | 4.6 | 5.0 | 5.0 | 5.1 | 4.7 | stripping | | | | | |
| 12 | v | stripping | | | | | | 4.6 | 4.6 | 3.9 | 4.8 | 4.8 | 4.0 |
| 13 | v | stripping | | | | | | 4.8 | 3.5 | 4.0 | 4.4 | 3.6 | 4.3 |
| 14 | v | 5.1 | 3.4 | 2.7 | 5.1 | 3.7 | 3.2 | 5.1 | 4.1 | 3.2 | 5.1 | 4.6 | 3.5 |
| 15 | v | 4.1 | 3.1 | 2.8 | 5.5 | 3.2 | 2.7 | 4.4 | 4.1 | 3.5 | 4.5 | 4.0 | 4.1 |
| 16 | v | stripping | | | | | | 5.1 | 4.8 | 4.0 | 5.0 | 4.5 | 4.1 |
| 17 | v | 4.9 | 4.3 | 4.4 | 5.1 | 4.7 | 4.5 | amputation | | | | | |
| 18 | v | 5.5 | 5.0 | 5.3 | 6.1 | 5.1 | 5.1 | 4.7 | nd | nd | 4.5 | nd | nd |
| 19 | v | stripping | | | | | | 4.8 | 3.4 | 3.8 | 4.8 | 3.3 | 3.5 |
| 20 | v | not clearly definable | | | | | | 6.3 | 4.0 | 4.5 | 5.5 | 4.2 | 4.5 |
| 21 | v | 5.8 | 4.7 | 4.1 | 5.7 | 5.3 | 4.2 | 3.1 | 3.4 | 2.1 | 2.0 | nd | nd |
| 22 | v | 12.0 | 9.8 | 9.2 | 11.5 | 9.6 | 9.3 | stripping | | | | | |
| 23 | v | stripping | | | | | | 4.6 | 3.7 | 3.6 | 4.6 | 3.9 | 3.4 |
| 24 | v | 5.8 | 7.4 | 3.8 | 6.0 | 6.8 | 4.0 | stripping | | | | | |
| 25 | p | 3.6 | 3.2 | 3.1 | 3.7 | 3.3 | 2.8 | 3.7 | 2.8 | 1.5 | 3.5 | 2.5 | 1.2 |
| 26 | p | 5.8 | 5.8 | 10.6 | 6.3 | 6.7 | 6.4 | 7.4 | 6.6 | 5.7 | 7.0 | 5.9 | 6.1 |
| 27 | p | 3.1 | 3.7 | 3.2 | 3.4 | 3.7 | 3.2 | 3.9 | 3.2 | 3.0 | 4.2 | 2.4 | 2.6 |
| 28 | -- | 5.3 | nd | nd | nd | nd | nd | nd | nd | nd | 3.0 | nd | nd |
| 29 | -- | 5.7 | 4.4 | ni | 5.1 | 2.1 | 3.7 | 6.5 | 5.7 | 5.7 | 6.5 | 5.4 | 5.8 |
| 30 | -- | 18.1 | 17.3 | 11.0 | 18.6 | 20.2 | 13.0 | stripping | | | | | |
| 31 | -- | 5.5 | 3.7 | 4.4 | 6.2 | 3.3 | 4.6 | 5.7 | ni | 3.4 | 6.0 | 4.2 | 3.4 |
| 32 | -- | 3.9 | 2.1 | 1.7 | 3.6 | 2.4 | 2.6 | 3.9 | 3.2 | 2.8 | 2.8 | 2.0 | 3.0 |
| 33 | -- | 2.9 | 1.9 | ni | 2.9 | 1.9 | 2.6 | 2.1 | 2.0 | 2.4 | 2.8 | 2.0 | 3.0 |
| 34 | -- | 3.9 | nd | nd | 4.0 | nd | nd | 3.9 | 3.5 | 4.6 | 3.7 | 3.4 | 4.2 |
| 35 | -- | 3.5 | 2.3 | 1.9 | 4.1 | 2.0 | nd | 3.8 | 2.2 | 2.5 | 4.3 | 2.1 | 2.9 |
| 36 | -- | stripping | | | | | | 11.4 | 14.3 | 11.7 | 11.2 | 13.1 | 14.2 |
| 37 | -- | stripping | | | | | | 5.9 | 3.7 | nd | 4.2 | 2.8 | nd |
| 38 | -- | 4.5 | 3.3 | 3.2 | 4.6 | 3.5 | 3.2 | 4.4 | 3.2 | 3.7 | 4.4 | 3.5 | 4.0 |

GSV = great saphenous vein

nd = not definable

ni = not included

v = vein

p = prosthetic graft
